# Reconciling Associations Between Minority Stress and Sexual Minority Romantic Relationship Functioning

**DOI:** 10.3389/fpsyg.2021.707058

**Published:** 2021-06-25

**Authors:** David Matthew Doyle, Lisa Molix

**Affiliations:** ^1^Washington Singer Laboratories, Department of Psychology, University of Exeter, Exeter, United Kingdom; ^2^Department of Psychology, Tulane University, New Orleans, LA, United States

**Keywords:** minority stress, social stigma, sexual minority, relationship functioning, meta-analysis

## Introduction

Cao et al. (2017) conducted a meta-analysis of the association between sexual minority stress and relationship well-being. Having published a similar meta-analysis (Doyle and Molix, 2015b), we were struck by a number of important similarities as well as critical differences in these two reviews, as other authors working on minority stress and sexual minority romantic relationship functioning have also been recently (e.g., Ballester et al., 2021; Vale and Bisconti, 2021). Here we aim to reconcile discrepant findings with regard to conceptual and methodological differences between the two reviews. Additionally, we aim to contribute to a broader discussion of understanding overlapping meta-analytic reviews (Siontis et al., 2013; Helfer et al., 2015).

In order to integrate findings from these two reviews, for illustrative purposes we began by conducting a second-order meta-analysis using the *psychmeta* package in R (Dahlke and Wiernik, 2018). For reasons discussed in detail later, we conducted a “bare bones” second-order meta-analysis using uncorrected first-order meta-analysis estimates weighted by the number of primary studies included (Schmidt and Oh, 2013), shown in Table 1. Overall, the combined estimate of the effect of minority stress on sexual minority relationship functioning across these two meta-analyses was small but statistically significant, *r* = −0.144, 95% CI (−0.157, −0.132), echoing the importance of understanding minority stress processes for research on sexual minority romantic relationships. We continue to refer to the results of our “bare bones” second-order meta-analysis as we discuss similarities and differences in the two reports.

**Table 1:** Results of “bare bones” second-order meta-analysis of associations between minority stress and relationship functioning.

	***k***	***r***	***SE***	**95% CI**
Overall minority stress	71	−0.144	0.006	−0.157, −0.132
Overall minority stress (excluding visibility management)	71	−0.152	0.005	−0.161, −0.143
Internalized stigma	58	−0.166	0.004	−0.173, −0.158
Perceived stigma	24	−0.102	0.009	−0.120, −0.085

*k = number of independent effect sizes, corrected covered area (CCA) = 0.396*.

## Conceptual Differences

### Inclusion of Visibility Management

In framing the research, both meta-analyses drew upon Meyer (2003) influential minority stress theory to conceptualize various stressors for sexual minorities. In addition to internalized stigma and perceived stigma, included in both reports, Cao et al. included visibility management as a third form of minority stress, a decision marking an important distinction between these two reviews. Past research on the implications of sexual orientation concealment (and identity management more broadly) for romantic relationships has revealed mixed results (Lavner, 2017; Vale and Bisconti, 2021). This is potentially because concealment is not *just a stressor*, but also a coping strategy by which sexual minorities can manage the stigma to which they are exposed (Talley and Bettencourt, 2011; Doyle and Molix, 2016b; Pachankis and Bränström, 2018). Therefore, we agree that it is sensible to examine the influence of visibility management on sexual minorities' romantic relationships, however these effects should not be collapsed into a broader umbrella of minority stress alongside internalized and perceived stigma.

In line with this reasoning, the estimate of the effect of visibility management presented by Cao et al. was not statistically significant and was almost equal to zero, *r* = 0.008, 95% CI (−0.043, 0.059). By including this variable as a form of minority stress alongside internalized stigma and perceived stigma, the overall estimate of the effect of minority stress on same-sex relationships becomes muddled and, in this case, attenuated. Thus, while we reported an overall association of small to moderate magnitude, *r* = −0.17, 95% CI (−0.20, −0.14), Cao et al. reported an overall association of substantially smaller magnitude, *r* = −0.090, 95% CI (−0.140, −0.040), with neither estimate contained in the other's confidence interval. While this difference may appear relatively trivial, it can lead to problematic characterizations regarding the average effect of minority stress (e.g., Cao et al. refer to this association as “quite small” on p. 1269). A combined estimate excluding visibility management from our second-order meta-analysis, *r* = −0.152, 95% CI (−0.161, −0.143), provides support for a relatively larger magnitude effect size, closer to that reported in our original meta-analysis. We do concur with Cao et al. in that visibility management is an important facet of sexual minority experience that may influence romantic relationships, but we recommend exploring its effects in a conceptually distinct way that acknowledges the agency of sexual minority individuals to negotiate their identities in confrontation with social stigma (Doyle and Molix, 2016b).

### Role of Perceived Stigma

Further drawing upon Meyer's minority stress model, both of these analyses used the proximal-distal distinction to hypothesize which forms of minority stress would be most damaging for same-sex relationships. Consistently, in both introductions, internalized stigma (also recently referred to as “internalized negativity” to reflect the process of coming to associate negative attitudes with one's own identity; Dyar et al., 2018) was highlighted as being particularly pernicious and problematic for relationship functioning. Confirming this hypothesis, both meta-analyses found evidence for moderation of effect sizes by minority stress type, and in both cases, estimates for effect sizes involving internalized stigma were larger than for effect sizes involving perceived stigma. Therefore, both reviews correctly emphasized the importance of understanding how internalized stigma contaminates romantic relationships for sexual minorities. However, a critical difference emerged in the point estimates for perceived stigma.

In our analysis, the effect of perceived stigma on relationship functioning was found to be statistically significant, *r* = −0.12, 95% CI (−0.16, −0.08), while in Cao et al.'s analysis it was not, *r* = −0.053, 95% CI (−0.108, 0.002). A combined estimate from our second-order meta-analysis, *r* = −0.102, 95% CI (−0.120, −0.085), confirms a small but deleterious association between perceived stigma and relationship functioning. A growing body of work (e.g., Doyle and Molix, 2014, 2016a) has been documenting the ways in which prejudice and discrimination negatively affect social relationships, including romantic relationships, for sexual minorities. While internalized stigma is undoubtedly important to social relationships, so too is perceived stigma, which damages relationships through pathways such as decreasing self-esteem (Doyle and Molix, 2014) and increasing negative affect (Vale and Bisconti, 2021). Moreover, internalized negativity is produced over time through exposure to prejudicial and discriminatory social systems and environments (Meyer, 1995; Williamson, 2000; Szymanski et al., 2008), which can sometimes even lead to a normalization of hegemonic stigmatizing attitudes regarding gender and sexual orientation (i.e., sexism, homonegativity) among sexual minorities themselves (López-Sáez et al., 2020). Although statistical significance of a point estimate should not determine whether an association is considered meaningful or not, we are concerned that portraying the association between perceived stigma and relationship functioning as smaller and therefore potentially less important than it truly is may impede work to change social structures and policies protecting sexual minorities from discrimination.

## Methodological Differences

### Number of Studies/Effect Sizes Included

Turning to methodological differences, as is common with overlapping meta-analyses on similar topics (Siontis et al., 2013), there were differences in the number of studies, and consequently effect sizes, included in these reports. With a notable difference in independent variables (excluding visibility management in ours), we identified 35 studies reporting 130 effect sizes; Cao et al. identified 32 studies reporting 179 effect sizes. Based on materials presented in Cao et al.'s supplemental tables, we were able to examine potential differences in study identification. Our review did not include 6 studies identified by Cao et al. that were published after our search had concluded as well as 6 studies that focused exclusively on visibility management. We were able to ascertain that 10 of the studies not identified by Cao et al. were unpublished dissertations and 1 was a book chapter (both considered “gray literature”), with only 5 published journal articles missing, potentially because they did not meet inclusion criteria. Although Cao et al. report in their methods that gray literature was included in their search strategy, exclusion or under-identification of gray literature in meta-analyses can bias effect size estimates (Conn et al., 2003). Moreover, because Cao et al. did not include a study inclusion flowchart (Moher et al., 2009) or a table of extracted effect sizes, we are unable to fully discuss and contrast methodological differences related to study/effect size inclusion decisions. Therefore, comparisons between the specific effect sizes included in the two reports are not possible, nor is a pooled omnibus meta-analysis (Schmidt and Oh, 2013).

### Inclusion of Regression Coefficients

Another critical methodological difference between these two meta-analyses involves the inclusion of effect size estimates from slopes in multiple regression analyses (i.e., from standardized regression coefficients, or betas). The inclusion of betas in meta-analysis, while sometimes done in practice, has recently been heavily criticized (e.g., Aloe, 2015). The key issue here is that these coefficients are adjusted for other relevant independent variables but there may or may not be consistency in what specific independent variables (or covariates) are included across studies. So, for example, one study might control for depressive symptomatology which is a correlate (and potentially product) of minority stress. Including depressive symptomatology in a multiple regression analysis might attenuate direct associations between minority stress and relationship functioning, thus attenuating effect size estimates in the meta-analysis and introducing spurious heterogeneity across studies.

### Moderation by Gender

An important similarity between these reviews is that both highlighted a lack of sample diversity (e.g., age, race) and methodological diversity (e.g., longitudinal, experimental) as major limitations of the literature. However, while Cao et al. found evidence for moderation by gender (with stronger effects for samples of sexual minority women and mixed gender samples relative to samples of sexual minority men), we did not find similar evidence. This difference may be due to the different number of studies identified by gender (we found equal numbers of studies with samples composed of sexual minority women and men while Cao et al. found more studies focusing on sexual minority women than men) or to a difference in the effect of visibility management by gender as we did not include this variable in our analyses. A weighted analysis might help to further shed light on this issue. Future research should also aim to clarify this point, as unraveling the intersection of gender identity and sexual orientation (including greater consideration of gender diversity) is vital for work in this area.

## Discussion

In summary, our conceptual and methodological review and “bare bones” second-order meta-analysis indicate that the overall association between minority stress and sexual minority romantic relationship functioning is likely to be small but substantial, particularly when excluding visibility management as a form of minority stress. There is need for greater nuance in research on how visibility management affects sexual minority relationships in future work, potentially distinguishing identity management strategies motivated by internalized negativity (López-Sáez et al., 2020; Ballester et al., 2021) as opposed to efforts at coping with prejudice and discrimination (Doyle and Molix, 2016b; Pachankis and Bränström, 2018). Furthermore, the association of relationship functioning with internalized stigma is of greater magnitude than with perceived stigma, but the deleterious role of perceived stigma in sexual minority romantic relationships is still meaningful and should not be overlooked by researchers. We recommend that future work on this topic continue to investigate the effects of not only internalized *and* perceived stigma, but also structural stigma. Research bridging multiple levels of analysis (e.g., intrapersonal, interpersonal, systemic) will be important in revealing a nuanced picture of the ways in which discriminatory social environments affect social relationships for sexual minority people (Doyle and Molix, 2015a).

Methodologically, the exclusion of gray literature and inclusion of standardized regression coefficients in meta-analyses on this topic is likely to bias estimates of the association between minority stress and sexual minority romantic relationship functioning. Therefore, we recommend that any future reviews on this topic involve a thorough and concerted search of unpublished work (including dissertations and theses), with reviewers contacting primary authors when necessary to determine unadjusted coefficients not reported in published studies. Finally, future research should aim to situate work in this area within the intersection of gender identity and sexual orientation, examining the possibility of differences in the (potentially multiplicative) effects of social stigma on sexual minority romantic relationships involving transgender and gender diverse people.

Overall, we are encouraged by the fact that research on sexual minority stress and relationship functioning seems to be growing. While we aimed to draw attention to some concerns with specific methodological and conceptual decisions, we are heartened to see continued work in this area building upon the research covered in our review. The results of our “bare bones” second-order meta-analysis integrate findings from these two reports, continuing to indicate an important role for minority stress in shaping romantic relationship functioning among sexual minorities and pointing toward directions for future work on this topic.

## Author Contributions

DD conducted the analyses and wrote the first draft of the manuscript. DD and LM contributed to manuscript revision, read, and approved the submitted version. All authors contributed to the article and approved the submitted version.

## Conflict of Interest

The authors declare that the research was conducted in the absence of any commercial or financial relationships that could be construed as a potential conflict of interest.
